# Comparative Efficacy and Safety of Mycophenolate Mofetil and Azathioprine in Combination with Corticosteroids in the Treatment of Lymphocytic Myocarditis

**DOI:** 10.3390/jcm12154913

**Published:** 2023-07-26

**Authors:** Olga Blagova, Ruslan Rud’, Evgeniya Kogan, Alexander Zaitsev, Alexander Nedostup

**Affiliations:** 1 Department of Faculty Therapy No.1, N.V. Sklifosovsky Institute of Clinical Medicine, I.M. Sechenov First Moscow State Medical University (Sechenov University), 6, B. Pirogovskaya St., 119992 Moscow, Russia; ruslancardio@yandex.ru (R.R.); avnedostup@mail.ru (A.N.); 2 Department of Pathology, N.V. Sklifosovsky Institute of Clinical Medicine, I.M. Sechenov First Moscow State Medical University (Sechenov University), 119992 Moscow, Russia; koganevg@gmail.com; 3 Department of Endovascular Methods of Diagnostics and Treatment, I.M. Sechenov First Moscow State Medical University (Sechenov University), 119992 Moscow, Russia; sir-auz@mail.ru

**Keywords:** lymphocytic myocarditis, endomyocardial biopsy, immunosuppressive therapy, azathioprine, mycophenolate mofetil

## Abstract

Aims: This paper aimed to study the efficacy and safety of mycophenolate mofetil (MM) in combination with corticosteroids in the treatment of lymphocytic myocarditis (LM) when compared to the standard combination of corticosteroids and azathioprine. Methods. The study included 50 adult patients (47.8 ± 10.8 y.o.) in a NYHA III functional class due to LM who were verified using endomyocardial biopsy. The main group included 29 patients who received MM at 2 g/day. The comparison group comprised 21 patients who received azathioprine at 150 [50; 150] mg/day. Both groups were administered with methylprednisolone. The average follow-up period was 30 [22; 35] months, but no less than 6 months. Results. The groups were comparable in the baseline parameters and standard drug therapy. In both groups, there was a comparable significant increase in the ejection fraction (from 30.6 ± 7.7% to 44.0 ± 9.4% vs. 29.2 ± 7.7% to 46.2 ± 11.8%, *p* < 0.001), and a decrease in systolic pressure in the pulmonary artery and the dimensions of the left ventricle and atrium. The frequency of death was two (6.9%) and two (9.5%), transplantation was one (3.4%) and one (4.8%) patient and the “death + transplantation” endpoint was three (10.3%) and three (14.3%) without differences between the groups. The presence of the parvovirus B19 in the myocardium in 6/5 patients did not affect the results. The incidence of infectious complications was comparable. The most severe infectious complications were pneumonia and fatal purulent encephalitis (both cases in the azathioprine group), leptospirosis meningitis (in the mycophenolate mofetil group). Conclusions. In the patients with LM, the combination of corticosteroids with MM at a dose of 2 g/day was at least no less effective than with azathioprine. There was a tendency toward a better tolerance using MM.

## 1. Introduction

The treatment of myocarditis remains one of the most challenging problems in cardiology and internal medicine. Its true frequency is unknown. In early studies during outbreaks of the Coxsackie virus infection, heart damage was assumed in 3.5–5% of patients. In the Global Burden of Disease Study, when assessing discharge records for the period from 1990 to 2013, only 22 cases of myocarditis were detected per 100,000 patients annually [1]. The difficulty in diagnosing myocarditis is that a reliable diagnosis can only be made with the help of endomyocardial biopsy (EMB), which is rarely used. There is no non-invasive “gold standard”. Due to the difficult-to-reach diagnosis, the treatment of myocarditis is often not conducted, which leads to a decrease in the LV systolic function, its remodeling with the dilatation of the heart, CHF, life-threatening ventricular arrhythmias and sudden death.

The verification of the diagnosis of myocarditis using EMB is necessary in order to conduct its differentiated therapy. Highly effective antiviral therapy does not exist (although when certain pathogens are identified, such therapy is justified and necessary). The basis for the treatment of myocarditis, especially its severe forms, is immunosuppressive therapy (IST). If the rare forms of severe myocarditis (granulomatous, eosinophilic, giant cell) are not included for the main morphological variant—lymphocytic myocarditis (LM)—the condition for IST is the absence of the viral genome in the myocardium. Recent studies also showed the effectiveness of IST in the presence of the parvovirus B19 genome in the myocardium [2,3].

The feasibility of IST in viral-negative LM was confirmed in the well-known TIMIC study of 2009 and in subsequent larger European single-center cohort studies [4]. It is important to emphasize that the same studies showed an unfavorable course of myocarditis in the absence of IST (in the control groups persistent LV systolic dysfunction and an increase in mortality were noted). The TIMIC study was also considered a reference in terms of the choice of the IST regimen. The standard protocol was maintained as the prescription of high doses of corticosteroids (1 mg/kg based on prednisolone) for one month, followed by a stepwise decrease in combination with azathioprine at 1–2 g/kg for a 6 month period. However, the use of high doses of corticosteroids, even for a limited time, is associated with the development of serious side effects. Azathioprine also exhibits side effects, which may require an early withdrawal of drugs and a search for alternative solutions.

However, unlike many other immune-mediated diseases, conventional alternative treatment regimens in myocarditis are completely absent. There are practically no specially planned studies using other cytostatics or targeted biological drugs. One of the very few drugs that has been used successfully for myocarditis in selected patients is mycophenolate mofetil (MM). It is a prodrug that rapidly metabolizes to mycophenolic acid—a selective, noncompetitive, reversible inhibitor of inosine monophosphate dehydrogenase, a de novo enzyme of the guanine nucleotide synthesis pathway—which is important for the proliferation of B and T lymphocytes. Additional effects of the drug include the appearance of apoptosis of T-lymphocyte clones activated by antigens, a decrease in the production of antibodies and the expression of adhesion molecules, the detection of leukocytes and monocytes in the foci of inflammation and the antifibrotic effect.

Transplantology and rheumatology remain the main niches for MM applications. In the only clinical study involving 20 patients with viral-negative LM, the effect of MM at a dose of 1 g/day was studied with a gradual increase in the dose after 4 weeks to 2–3 g/day. After 6 months of therapy, there was a significant increase in the LV EF, a decrease in its volume and the levels of troponin T and NT-proBNP and an improvement in the functional status of the patients [5]. A case for the successful use of MM for the treatment of giant cell myocarditis resistant to standard IST has also been published [6].

These data inspire optimism and provide a foundation for a more detailed study of using MM in myocarditis. However, MM monotherapy cannot be considered to be optimal. Studies of MM in combination with corticosteroids have not been carried out, and there are no studies that are comparable with the standard treatment regimen. Additionally, recommendations for the use of MM in LM have not been developed. Therefore, the outcomes of this study are highly relevant.

The aims of this paper are to study the efficacy and safety of MM (in combination with corticosteroids) in the treatment of LM compared to the standard combination of corticosteroids and azathioprine.

## 2. Methods

The study included fifty patients with LM (38 men and 12 women, with a mean age of 47.8 ± 10.8 years).

The study inclusion criteria were patients aged 18 years and older, an EMB diagnosis of LM (active or borderline, according to the Dallas criteria, virus-negative, except for parvovirus B19), a CHF 2-4 NYHA functional class (FC), LV dysfunction persisting after 2 months of optimal drug therapy (LV EDS more than 5.5 cm, EF less than 50%) and the presence of written informed consent of the patient to participate in the study.

The study exclusion criteria included a history of myocardial infarction, chronic coronary syndromes with hemodynamically significant stenosis of the coronary arteries (from 70% or more), congenital heart defects, infective endocarditis, thyrotoxic or hypertensive heart (LV hypertrophy more than 14 mm), hypertrophic cardiomyopathy, sarcoidosis, diffuse connective tissue diseases and systemic vasculitis, lymphoproliferative diseases, anthracycline chemotherapy and cardiac surgery less than 2 months before the start of the study.

### 2.1. Research Methods

All the patients underwent physical and standard laboratory tests, an investigation of the anti-heart antibodies (to antigens of cardiomyocyte nuclei, endothelium, cardiomyocytes, fibers of the cardiac conduction system, smooth muscles) in the blood using the method of indirect immunofluorescence, echocardiography (EchoCG), Holter monitoring of an electrocardiogram (ECG), coronary angiography (*n* = 26), multispiral computed tomography (MSCT) of the heart (*n* = 26) and magnetic resonance imaging (MRI) of the heart (*n* = 22). To exclude systemic viral infections, a blood test was performed using the polymerase chain reaction (PCR) method.

**Figure 1:**
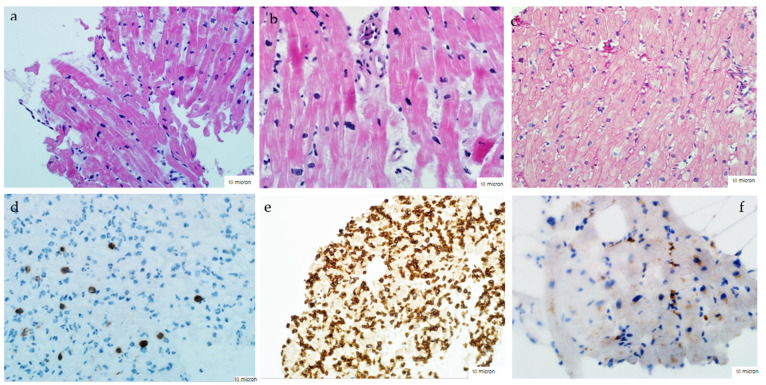
Morphological signs of lymphocytic myocarditis according to the histological (upper row) and immunohistochemical (lower row) studies of the biopsy specimens of the right ventricular myocardium. Hematoxylin-eosin staining (**a**,**b**), according to Van Gieson (**c**), immunohistochemical preparations with antibodies to CD3 ((**d**), more than seven positive cells), CD45 ((**e**), more than 14 positive cells), CD68. Interstitial (**a**,**d**,**e**) and perivascular (**b**) lymphocytic and macrophage (**f**) infiltration; perivascular (**b**) and small focal (**c**) fibrosis.

The diagnosis of myocarditis in all the patients was verified using EMB of the right ventricle using Cordis biopsy forceps. Three to five specimens of the myocardium were collected, followed by histological (staining with hematoxylin-eosin and Van Gieson), immunohistochemical (with antibodies to CD3, CD45, CD20 and CD68) and virological (PCR with determination DNA of parvovirus B19, adenoviruses, enteroviruses, complete spectrum of viruses of the herpes group) research. The diagnosis of active/borderline myocarditis was based on the Dallas criteria supplemented by the immunohistochemical criteria (Figure 1).

### 2.2. Study Designn

The study was a single-center prospective cohort study. After confirming the diagnosis of LM, excluding viral infections (with the exception of parvovirus B19) and evaluating the other inclusion and exclusion criteria, the investigator divided the patients into one of two groups—the main group or the comparison group. The main group included 29 patients who received methylprednisolone at an initial dose of 24–40 mg/day orally for 1 month, followed by a decrease to a maintenance dose (4–8 mg/day) in combination with MM 2 g/day orally. Of these, 23 patients received MM as the first drug, six more were prescribed MM after azathioprine (due to the lack of efficacy or side effects of the latter). The comparison group included 21 patients who received methylprednisolone at an initial dose of 24–40 mg/day per os, followed by a reduction to a maintenance dose (4–8 mg/day) in combination with azathioprine 1–2 mg/kg per os (100–150 mg/day). All the patients also received standard CHF therapy, which included beta-blockers, angiotensin-converting enzyme inhibitors (ACE inhibitors) or sacubitril–valsartan and mineralocorticoid receptor antagonists (AMR).

The average fallow-up period was 30 [22; 35] months, but no less than 6 months for each patient. Every six months and at the end of the observation, a follow-up examination was conducted, which included an assessment of the functional status, standard blood tests, level of anti-heart antibodies, EchoCG and Holter monitoring.

The study endpoints were death/heart transplantation and the frequency of withdrawal/replacement of the drug due to side effects. The dynamics of the NYHA FC and the main structural and functional parameters of the heart (EF, including an increase of 10% or more; LV dimensions; PASP) were also assessed.

A statistical analysis was conducted using the SPSS software package version 23. The quantitative characteristics are presented as M ± δ (mean ± one standard deviation) or as a median indicating the first and third quartiles. The normality was assessed using the Kolmogorov–Smirnov test, the significance of the differences was assessed using the Student’s test, χ^2^ or Fisher’s test, Mann–Whitney test or Wilcoxon tests. To assess the survival rate, Kaplan–Meier curves were constructed. The differences were considered statistically significant at *p* < 0.05.

The study was approved by the local ethics committee of Sechenov University (protocol No. 33–20 of 25 November 2020). All the patients provided voluntary informed consent for EMB and the subsequent IST. ClinicalTrials.gov Identifier: NCT05237323.

## 3. Results

The comparative clinical characteristics of the patients in both groups are presented in Table 1. There were no significant differences in the baseline characteristics between the groups. The groups were completely comparable in age, sex, NYHA FC, the degree of systolic dysfunction, the duration of the symptoms and the size of all heart chambers. The significantly shorter follow-up period in the main group was due to the predominant appointment of MM during the last 1–1.5 years, when azathioprine was nearly completely absent in the pharmacy, as well as the transition to the group of six patients who had previously received azathioprine.

**Figure 2:**
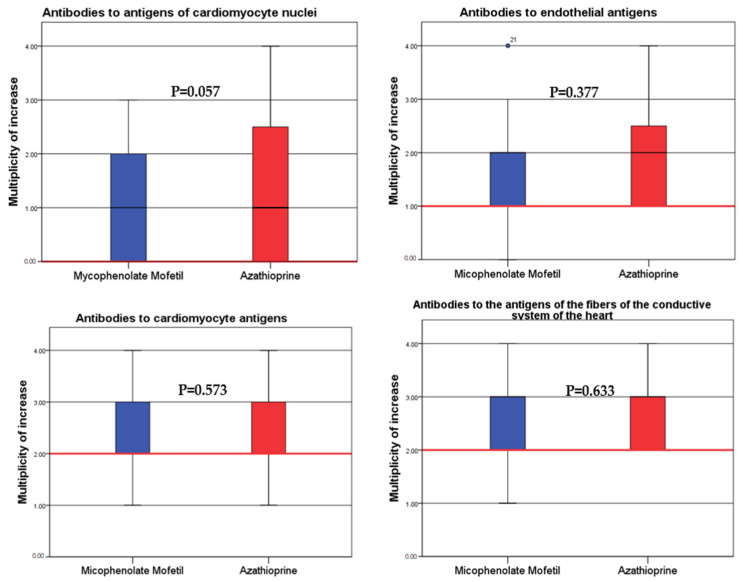
Comparison of the baseline level of the anti-heart antibodies in the patients of the main group (MM) and the comparison group (azathioprine).

When comparing the immune activity of myocarditis, which was assessed by the level of anti-heart antibodies, there were no significant differences between the groups (Figure 2).

In addition to IST, the patients received standard cardiotropic (for CHF), antiarrhythmic and anticoagulant therapy (Table 2). In several cases, an ACE inhibitor in both groups was replaced by ARNI. Along with drug therapy, some patients underwent interventional treatment. An implantable cardioverter defibrillator (ICD) was implanted in one patient from the main group and in three from the comparison group. Three CRT-D devices were implanted in the comparison group.

The ordinate shows the ratio of the increase in the antibody titer from 1—1:40, 2—1:80, 3—1:160 and 4—1:320. The red horizontal line represents the normal value.

### 3.1. Dynamics of the Main Structural and Functional Parameters of the Heart and the Level of Anti-Heart Antibodies in the MM and Azathioprine Treatment Groups

**Figure 3:**
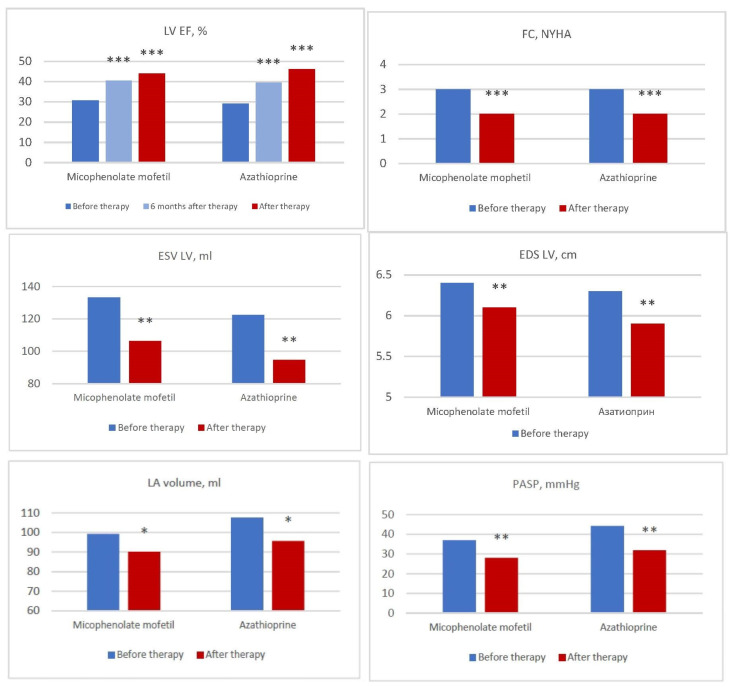
Dynamics of the NYHA FC and basic echocardiography parameters in the patients with myocarditis in the groups of mycophenolate mofetil and azathioprine. LV EDD—end-diastolic size of the left ventricle, LV EDV—end-systolic volume of the left ventricle, LV ESV—end-systolic volume of the left ventricle, LVEF—left ventricular ejection fraction, FC—functional class, LA—left atrium, PASP—systolic pressure in the pulmonary artery, NYHA—the New York Heart Association Functional Classification; *—*p* < 0.05, **—*p* < 0.01, ***—*p* < 0.001.

In both groups, there was a significant and comparable decrease in the NYHA FC (Figure 3), which was accompanied by a subjective improvement in the patients’ well-being.

EchoCG was evaluated every six months until the end of the observation period or until an unfavorable outcome occurred. Depending on the dynamics of the EF, the response to treatment was determined to be excellent with an increase in the EF by 10% or more, good with an increase of 5–9% and lack of response with a lesser degree of increase or decrease in the EF.

Comparable highly significant (*p* < 0.001) positive dynamics in the EF was observed in both groups after 6 months from the start of the treatment and by the end of the study (Figure 3). The overall increase in the EF was 13% in the MM group and 17% in the azathioprine group by the end of the follow-up period. At the same time, an excellent response to treatment (an increase in the EF by 10% or more) was noted in 55.2% and 61.9% of the patients, a good response (by 5–9%) in 24.1% and 9.5% of the patients and a lack of response (an increase of less than by 5% or a decrease in the EF) in 10.3% and 19.0% of the patients, respectively. In both groups, there was also In identical significant (*p* < 0.01) decrease in the PASP, as well as the LV EDS, its EDV and the volume of the left and right atria (Figure 3).

The dynamics of the anti-heart antibody titers was assessed as a result of the treatment. The titers of all the types of antibodies decreased in both groups. However, by the end of the observation period, a significant decrease was observed in the titers of antibodies to antigens of cardiomyocytes, and endothelium and fibers of the conducting system were noted only in the MM treatment group (*p* < 0.05).

The comparison of the final structural and functional parameters in the MM and azathioprine treatment groups did not reveal significant differences (Table 3).

### 3.2. Assessment of the Outcomes (Study Endpoints)

In conclusion, we compared the main outcomes in the patients with myocarditis in the MM and azathioprine treatment groups.

**Figure 4:**
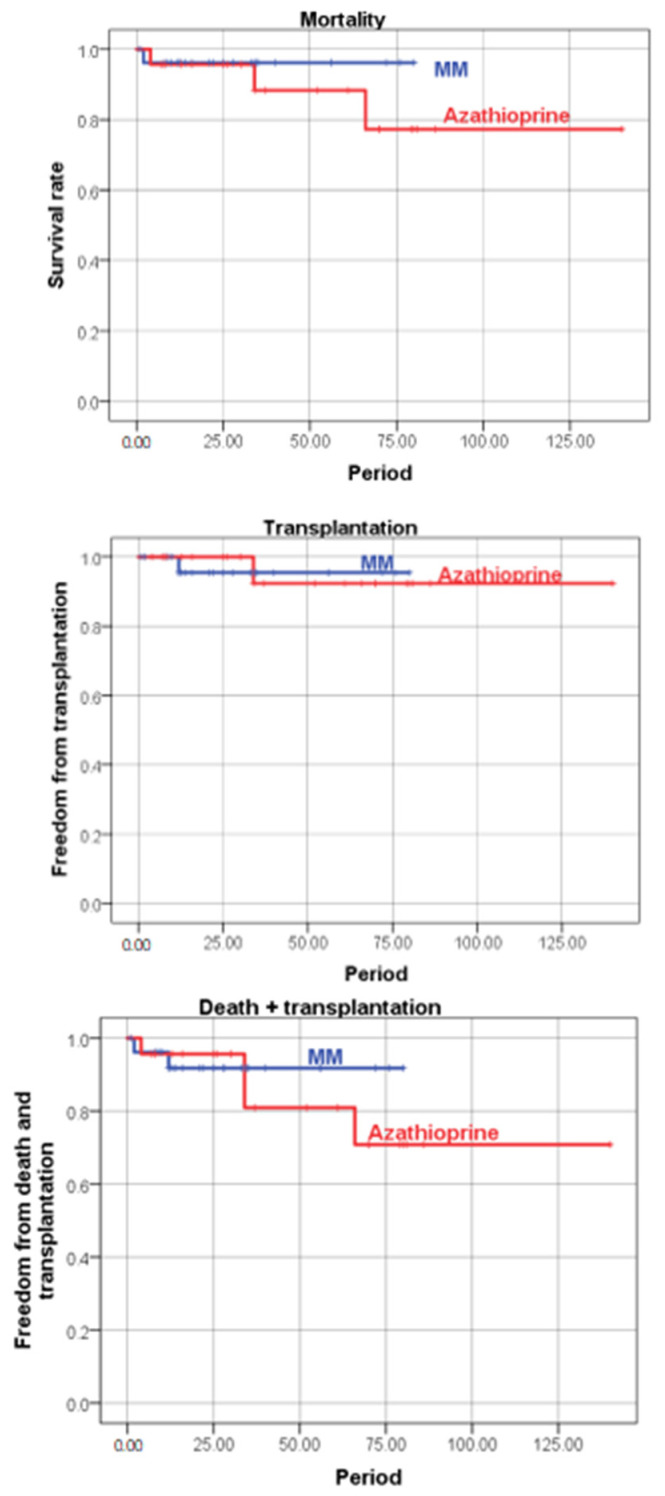
Comparison of the major endpoints in the mycophenolate mofetil (MM) and azathioprine treatment groups.

Mortality in the MM group was 6.9% (*n* = 2) and 9.5% in the azathioprine group (*n* = 2). By the end of the study, one heart transplant was successfully performed in each group due to end-stage CHF. There were no differences between the groups in the frequency of deaths, transplantations and the death + transplantation rate (Table 4, Figure 4).

#### Kaplan–Meier Curves

The causes of death were advanced CHF, infectious complications (the azathioprine treatment group) and sudden arrhythmic death (the MM treatment group). One of the deceased patients independently conducted a course of fasting in order to reduce weight (possible electrolyte disorders). The second case of sudden death developed in a patient with frequent ventricular premature beats, which persisted regardless of amiodarone and beta-blockers. The need for ICD implantation was considered, but death occurred within the first month of treatment (when it was too early to make a final decision).

The presence of the parvovirus B19 genome in the myocardium did not affect the immediate results of the treatment (the degree of improvement in the structural and functional parameters did not differ from that in the virus-negative patients) or the outcomes of the disease (endpoints). In the parvovirus-negative patients, by the end of the follow-up period, there was a significant decrease in the antibody titers to antigens of endothelium, cardiomyocytes and fibers of the conducting system, which was not observed in the virus-positive patients.

### 3.3. Comparison of the Safety of MM and Azathioprine Therapy

The subjective tolerance of cytostatics in both groups was satisfactory. All the emerging side symptoms were due to corticosteroid therapy and were reversible (regressed as the dose was reduced to a maintenance dose).

According to the study design, six patients, even before they were included in the comparative analysis, were transferred from azathioprine therapy to MM administration due to the insufficient efficacy of the latter (three patients retained high titers of anti-heart antibodies and a moderate degree of systolic dysfunction) or the development of serious cytopenia (mainly neutropenia and lymphopenia in combination with moderate anemia in three patients). In all the cases, the side effects of azathioprine completely regressed, which made it possible to begin MM therapy no earlier than one month after the discontinuation of azathioprine.

During the observation period in both groups, no cases of cytopenia required a discontinuation of the drugs. Infectious complications were recorded in both groups and were represented mainly by flu and acute bronchitis (in almost all the patients, no more than 1–2 times a year). For the period of a fever and the next 2–4 weeks, the cytostatic was canceled with the resumption of its administration after this period. Instead, antibacterial therapy was performed. In one case (in the azathioprine group), pneumonia developed.

Leptospirotic meningitis was successfully cured in a 64-year-old patient from the MM group, and a fatal purulent encephalitis of unknown etiology in a 56-year-old patient who received azathioprine. The deceased was diagnosed with lymphopenia, which could have been a consequence of coronavirus infection (no PCR or computed tomography was confirmed), developed sepsis and a side effect of azathioprine. The meningitis survivor suffered six months before the start of COVID-19 treatment and MM was completely canceled. In nine more patients, cytostatics were canceled by the end of the observation, taking into account the stable positive effect.

## 4. Discussion

The main result of this study was the full comparability of the results of LM therapy with both MM and azathioprine in combination with corticosteroids and the complex therapy of CHF. The explanation for the similar effectiveness of drugs was seen in the common mechanisms of their actions, specifically the inhibition of the purine pathway and the suppression of cell proliferation. Direct comparisons of this kind have never been conducted before, and the effectiveness of the combination of MM with corticosteroids in the treatment of LM has not been investigated. The study shows for the first time the high efficiency of the combination of MM with medium doses of steroids in the treatment of MM. According to its clinical characteristics, this myocarditis should be considered as subacute or chronic (the average duration of the onset of symptoms was 11 [5; 20] months), and virus-negative or positive for parvovirus B19.

Despite the absence of randomization and a special selection of pairs according to the “case-control” principle, the patients of both groups were completely comparable in their initial characteristics (functional status, EchoCG parameters, anti-heart antibodies). The lack of randomization was primarily associated with long-term interruptions in the production of azathioprine and its analogs in Russia in 2018–2020. Therefore, MM was predominantly prescribed during these years. The follow-up period in the main group was somewhat shorter than the azathioprine group. Nevertheless, in the MM group, it exceeded 1 year (on average 16 months), which can be considered more than sufficient to assess the efficacy and safety. For example, in the TIMIC study, the follow-up was conducted for 6 months.

Another characteristic of myocarditis in the patients included in this study was the severity of its course and the absence of spontaneous improvement. Despite the long term (at least 2 months) for the maximum possible therapy for CHF, all the patients at the time of inclusion persisted CHF symptoms (level 3 NYHA FC), systolic dysfunction (average EF is 30%) and dilatation of the heart chambers. There were undoubted indications for conducting IST.

An undoubted positive effect in both groups was proved in relation to the main structural and functional parameters, namely the LV EF, its size, left atrial volume (the dynamics of LV EDV was also positive but not significant), PASP and mitral regurgitation. When treated with azathioprine, more patients exhibited a poor effect of therapy (in 19% of them EF increased by less than 5% or decreased compared to 10% in the MM group), which may become significant with a larger number of patients. However, according to the results of the study, we still have no reason to discuss a higher efficiency of MM. The longer follow-up period in the azathioprine group may have also played a role.

By the end of this period, a significant decrease in the titers of the anti-heart antibodies (with the exception of the antibodies to the nuclei of cardiomyocytes) was noted only in the MM treatment group. One can think of a partial loss of the effect of azathioprine and corticosteroids during long-term (average 34 months) treatment with a gradual dose reduction. However, an insufficient decrease in the antibody titers (in combination with an insufficient increase in the EF) in a small number of patients required the replacement of azathioprine with MM. Therefore, there was a more pronounced ability of MM to suppress the production of autoantibodies. The literature contains data on the effective suppression of the humoral immune response in the treatment of MM of other autoimmune diseases (autoimmune hepatitis [7]). However, we could not identify special comparative studies on the effects of antibody production.

On the other hand, the presence of the parvovirus B19 genome in the myocardium had some negative effects on the decrease in the antibody titers. These data required verification and potentially reflected the ability of the virus to induce a more pronounced autoimmune response. However, the presence of the virus did not affect the final results of treatment. The role of parvovirus B19 in the genesis of myocarditis was not clear. It was found with a high frequency not only in the myocardium of the patients with a variety of heart diseases but also in most other organs and tissues of an adult. Many copies of the virus, however, is not always evidence of its etiological role. The previously mentioned clinical studies showed a comparable efficacy of the standard IST regimen in parvovirus-positive and viral-negative patients [2]. The CAPASITY study after 6 months of IST showed a significant improvement in LV systolic function, the disappearance of inflammatory infiltrates according to repeated EMB (with an unchanged number of copies of parvovirus B19), regardless of the presence of the virus or the degree of the viral load [3]. Our study showed the effectiveness of MM regardless of the presence of parvovirus and did not provide ground to fear the appointment of both MM and azathioprine in patients with parvovirus-positive LM.

The rates of death and transplantation were also completely comparable in both groups. It should be noted that both deaths in the MM treatment group were arrhythmic and could probably have been prevented by using ICD implantation. However, none of the patients had absolute indications (in the first, the EF exceeded 35%, and in the second, treatment for myocarditis had only just begun). The cases of heart transplantation in both groups were due to an insufficient effectiveness of complex therapy, which could not prevent the development of terminal CHF within a period of one to four years. However, in both patients, the genetic component of myocardial dysfunction (increased LV trabecularity on the verge of a non-compact myocardium in one patient) did not reach an optimal therapeutic effect and cannot be ruled out.

The global experience in the use of MM, including in comparison with other immunosuppressive agents, extends mainly to systemic immune diseases in which numerous comparative studies have been conducted. Thus, MM showed an equal efficacy with azathioprine in the treatment of interstitial lung diseases (the frequency of the MM side effects was significantly lower—33.3% vs. 13.6% [8]). In systemic lupus erythematosus, there is accumulating evidence of a lower risk of exacerbations using MM treatment compared to azathioprine, which was explained by a more pronounced suppression of B-lymphocyte proliferation [9]. In such aggressive diseases, such as ANCA-associated necrotizing vasculitis, MM in several studies showed an inferior efficiency compared to the main cytostatic agent cyclophosphamide [10]. It is not uncommon to administer MM in patients with insufficient effects to standard IST (for example, with autoimmune hepatitis).

There was a similar experience in relation to one of the most severe forms of autoimmune myocarditis—giant cell [6]. The diagnosis was made using EMB. From the systemic manifestations, there was ulcerative colitis. The extreme degree of systolic dysfunction (EF 6%) required the implantation of a ventricular auxiliary circulatory system and pulse therapy with methylprednisolone followed by the administration of prednisolone 50 mg/day, cyclosporine 50 mg/day and intravenous immunoglobulin, with the standard therapy for CHF. Due to the persistence of dysfunction (EF 15%), MM 1.5 g/day was added to the treatment with a gradual increase in the dose to 3 g/day. After 25 months, the EF reached 45%, and the functional status significantly improved. The case of the successful addition of MM to prednisolone has also been described in eosinophilic myocarditis [11].

However, we note once again that there are practically no targeted clinical studies that have studied the efficacy and safety of MM in myocarditis (in the form of monotherapy, in combination with corticosteroids, or in comparison with other IST regimens). MM is mentioned, as a rule, in the context of the treatment of myocarditis in systemic immune diseases [12] includes sarcoidosis with cardiac involvement [13]. In a recent study involving 77 patients, a comparison between monotherapy and corticosteroids and their combination with MM showed a more pronounced effect of combination therapy in combination with its better tolerance (due to lower doses of corticosteroids).

There was only one study on the effectiveness of MM monotherapy for LM in humans [5]. It included 20 patients who received MM 1 g/day with a gradual increase in the dose after 4 weeks to 2–3 g/day with systemic autoimmune disease of connective tissue or virus-negative LM that was resistant to standard IST. After 6 months of therapy, the LV EF significantly increased from 54% (44–61%) to 57% (50–61%), the LV EDV decreased from 135 ± 50 mL to 114 ± 38 mL, the troponin T level decreased from 50.5 to 12.0 ng/L, the NT-proBNP decreased from 257.0 to 79.5 ng/L and the functional status improved (initially 2–3 FC). There were no cases of drug withdrawal.

Our study initially included more severe patients (the average EF was about 30%). IST was complex in nature (cytostatic in combination with steroids), which probably made it possible to obtain a more pronounced positive clinical effect. An important difference in our study was also the isolated nature of LM (patients with systemic diseases were excluded). Among the markers of autoimmune activity, there was only an increase in anti-heart antibodies. Thus, our data allowed us to extend the experience of using MM beyond the framework of systemic immune pathology.

Our results provided a foundation to recommend the combination of MM with corticosteroids both as a starting LM therapy and in cases of insufficient efficiency, intolerance to the standard regimen (azathioprine + steroids), or if azathioprine is unavailable. Note that all the cases of cytopenia that developed during treatment with azathioprine and even before the start of the study required replacing azathioprine with MM. Therefore, we did not estimate the frequency of this side effect. These complication developed several months and even years after the start of azathioprine administration, possibly due to a planned reduction in the dose of steroids, which, as is well known, have a stimulating effect on the bone marrow.

There were no significant differences in the incidence of infectious complications (they were mainly of the nature of flu). We did not observe any other side effects of MM reported in the literature (dyspepsia due to suppression of epithelial proliferation, increased levels of hepatic transaminases, amylase or herpetic infections, which regressed with a decreasing dose [14]). Considering the best possible safety profile of MM, its appointment can be considered with difficult control over blood parameters in older, comorbid patients. However, further observation is necessary. So far, it has not been possible to identify any predictors of MM efficiency.

The current study does not yet provide sufficient evidence to favor a particular drug as a first-line therapy. MMF may be appropriate for highly immune active myocarditis (high titers of anti-heart antibodies) since it was effective in a number of patients with ineffective azathioprine. At the same time, azathioprine as a better proven drug may be preferred in patients with an increased risk of infectious complications (elderly, patients with a history of tumors, etc.). However, serious infectious complications have also occurred in the azathioprine group. These assumptions need to be verified in further studies.

## 5. Conclusions

In patients with moderate and severe virus-negative and parvovirus-positive lymphocytic myocarditis, the combination of moderate doses of corticosteroids with mycophenolate mofetil at a dose of 2 g/day was at least no less effective than the standard IST regimen (steroids with azathioprine). In the absence of baseline differences in all the basic structural and functional parameters of the heart and a shorter follow-up period, the MM group showed a tendency towards a more pronounced decrease in the anti-heart antibody titers in combination with better tolerance (no cases of cytopenia). The presence of the parvovirus B19 genome in the myocardium did not affect the immediate results and outcomes of the treatment. MM in combination with corticosteroids can be recommended as an alternative regimen for IST LM, both as primary therapy and when azathioprine is intolerant or unavailable.

## 6. Research Limitations

The limitations of the study were the relatively small number of patients (though comparable with the TIMIC study) and the absence of randomization due to objective reasons (the groups were completely comparable in terms of the baseline parameters).

## Figures and Tables

**Table 1:** Initial structural and functional parameters in the patients of the main group and the comparison group.

Initial Parameters	Mycophenolate Mofetil (*n* = 29)	Azathioprine (*n* = 21)	Significance of Differences (*p*)
Age	46.5 ± 11.9 years	49.8 ± 8.9 years	0.273
Sex	M 22 (75.9%); F 7 (24.1%)	M 16 (76.2%); F 5 (23.8%)	0.524
NYHA class	3 [3; 3]	3 [3; 3]	0.660
Duration of symptoms	10 [3.5; 9.0] month	9 [5.5; 31.5] month	0.564
EDS LV	6.4 ± 0.6 cm	6.3 ± 0.5 cm	0.448
EDV LV	191.6 ± 53.1 mL	173.4 ± 46.8 mL	0.345
ESV LV	133.3 ± 42.7 mL	122.5 ± 38.2 cm	0.420
LVEF	30.6 ± 7.7%	29.2 ± 7.7%	0.554
LA volume	99.2 ± 29.4 mL	107.6 ± 25.9 mL	0.069
RA volume	73.2 ± 28.9 mL	85.7 ± 26.1 mL	0.072
RV size	3.3 ± 0.6 cm	3.4 ± 0.5 cm	0.795
PASP	37 ± 12.5 mm Hg	44.2 ± 10.1 mm Hg	0.059
Parvovirus B19 in the myocardium	6 (20.7%)	5 (23.8%)	0.795
Follow-up period	28 [14; 34] month	34 [25; 61] month	0.044

FC—functional class, CHF—chronic heart failure, NYHA—New York Heart Association, LV ED—end-diastolic size of the left ventricle, EDV—end-systolic volume, CEV—end-systolic size, EF—ejection fraction, LA—left atrium, RA—right atrium, RV—right ventricle, PASP—systolic pressure in the pulmonary artery, M—men, F—female.

**Table 2:** Drug and interventional therapy in the main group and the comparison group.

Therapy	Mycophenolate Mofetil	Azathioprine	Significance of Differences (*p*)
Methylprednisolone (dose)	28 [24; 32] mg/day	24 [24; 24] mg/day	0.997
Cytostatics (dose)	2 g/day	150 [50; 150] mg/day	-
Beta-blockers	93.1%	95.2%	0.756
ACE inhibitors	67.9%	81%	0.309
ARNI	37.9%	9.5%	0.010
AMR	79.3%	90.5%	0.692
Amiodarone	44.8%	76.2%	0.028
Anticoagulants	48.3%	33.3%	0.296
ICD	1 (3.4%)	3 (14.3%)	0.151
CRT-D	0	3 (14.3%)	0.098

ACE—angiotensin-converting enzyme, AMR—mineralocorticoid receptor antagonists, ICD—implantable cardioverter defibrillator, CRT-D—cardiac resynchronization device with defibrillator function.

**Table 3:** The final structural and functional parameters in the patients of the main group and the comparison group.

Final Parameters	Mycophenolate Mofetil (*n* = 29)	Azathioprine (*n* = 17)	Significance of Differences (*p*)
NYHA class	2 [1; 2]	2 [1; 2]	0.777
LV EDS	6.1 ± 0.8 cm	5.9 ± 0.7 cm	0.304
LV EDV	187.9 ± 63 mL	165 ± 61.5 mL	0.207
LV ESV	106.4 ± 43.8 mL	94.8 ± 46.8 mL	0.288
LV EF	44.0 ± 9.4%	46.2 ± 11.8%	0.459
LA volume	90.0 ± 44.7 mL	95.7 ± 48.9	0.876
RA volume	64.0 ± 28.1 mL	82.7 ± 62.5 mL	0.383
RV size	3.1 ± 0.4 cm	3 ± 0.6 cm	0.370
PASP	28.0 ± 7.8 mm Hg	31.8 ± 12.5 mm Hg	0.361
MR	1.5 [1; 2]	1.5 [1; 2]	0.285

FC NYHA—classification according to the functional class of the New York Heart Association Functional Classification; LV EDS—end-diastolic size of the left ventricle, LV EDV—end-systolic volume of the left ventricle, LV ESV—end-systolic volume of the left ventricle, LVEF—left ventricular ejection fraction, FC—functional class, LA—left atrium, RA—right atrium, RV—right ventricle, PASP—systolic pressure in the pulmonary artery, MR—mitral regurgitation.

**Table 4:** Rate of study endpoint achievement in the mycophenolate mofetil and azathioprine treatment groups.

Endpoints	Mycophenolate Mofetil	Azathioprine	Significance of Differences (*p*)
Death	2 (6.9%)	2 (9.5%)	0.738
Transplantation	1 (3.4%)	1 (4.8%)	0.817
Death + transplantation	3 (10.3%)	3 (14.3%)	0.675

## Data Availability

Not applicable.

## Abbreviations

MM—mycophenolate mofetil, CHF—chronic heart failure, EF—ejection fraction, PASP—pulmonary artery systolic pressure, EDS—end-diastolic size, EDV—end-diastolic volume, ESV—end-systolic volume, EMB—endomyocardial biopsy, IST—immunosuppressive therapy, LM—lymphocytic myocarditis, LV—left ventricle, FC—functional class, EchoCG—echocardiography, ECG—electrocardiogram, MSCT—multispiral computed tomography, MRI—magnetic resonance imaging, PCR—polymerase inhibitor, AMR—mineralocorticoid receptor antagonists, ICD—implantable cardioverter defibrillator.
